# Antisense lncRNA NNT-AS1 promoted esophageal squamous cell carcinoma progression by regulating its sense gene NNT expression

**DOI:** 10.1038/s41420-022-01216-w

**Published:** 2022-10-21

**Authors:** Xianglong Pan, Qi Wang, Yue Yu, Weibing Wu, Liang Chen, Wei Wang, Zhihua Li

**Affiliations:** Department of Thoracic Surgery, The First Affiliated Hospital of Nanjing Medical University, Nanjing, China

**Keywords:** Long non-coding RNAs, Oncogenes

## Abstract

Antisense lncRNAs were endogenous productions from the antisense strand of coding genes and were transcribed in the reverse direction of the sense gene. The purpose of this study was to evaluate the roles and functions of antisense lncRNAs in esophageal squamous cell carcinoma (ESCC). Differentially expressed antisense lncRNAs were initially screened based on transcriptome data of 119 paired ESCC samples in GSE53624 and were further validated in 6 paired ESCC samples from our institution. Log-rank test was adopted to identify ESCC prognosis-associated lncRNAs. Finally, functional assays were performed to reveal the functions of our identified antisense lncRNAs. In total, 174 antisense lncRNAs were differentially expressed in both GSE53624 and JSPH databases. Five of them were significantly associated with ESCC prognosis (NNT-AS1, NKILA, CCDC18-AS1, SLCO4A1-AS1, and AC110619.1). Of note, NNT-AS1 showed the most significant association with ESCC prognosis. The upregulation of NNT-AS1 was further confirmed in ESCC cells. Knockdown of NNT-AS1 inhibited ESCC cell proliferation, migration, promoted ESCC cells apoptosis, and induced cell cycle arrest in the G2/M stage. NNT-AS1 expression significantly correlated with its sense gene NNT. As expected, NNT-AS1 knockdown suppressed NNT expression. Inhibition of NNT repressed ESCC cell proliferation and migration, and accelerated ESCC cell apoptosis. Overexpression of NNT could rescue the suppressed proliferation and migration of ESCC cells induced by the silencing of NNT-AS1. In terms of mechanism, NNT-AS1 served as a competing endogenous RNA to sponge the miR-382-5p, which could inhibit NNT expression. Pathway enrichment analysis and western blot assay indicated that NNT-AS1 and NNT could regulate the cell cycle pathway. In conclusion, antisense lncRNA NNT-AS1 facilitated ECSS progression by targeting its sense gene NNT through sponging miR-382-5p. This study provided us with a deeper insight into the roles of antisense lncRNAs in ESCC and identified novel potential therapeutic targets.

## Introduction

Esophageal cancer is one of the most diagnosed and deaths-caused malignant tumors around the world. As estimated, there were about 604 thousand new esophageal cancer cases (ranked seventh) and 544 thousand new deaths caused by esophageal cancer (ranked sixth) in 2020 [1, 2]. In contrast to the epidemic of esophageal adenocarcinoma in European countries, Esophageal squamous cell carcinoma (ESCC) is the most prevalent subtype in eastern Asia, eastern and southern Africa [3]. The prognosis of ESCC is poor with a five-year overall survival rate less than 30% in most countries [4, 5]. Therefore, doctors and researchers have to find novel diagnostic and therapeutic targets for ESCC.

Long non-coding RNAs (lncRNAs) could contribute to the development and progression of malignant tumors through multiple mechanisms [6, 7]. LncRNAs can be classified into several categories based on their positions relative to protein-coding regions: long intergenic noncoding RNAs, natural antisense transcripts, overlapping transcripts, bidirectional lncRNAs, and sense intronic lncRNAs [8]. Antisense lncRNAs are endogenous productions in nature that formed from the antisense strand of coding genes and transcribed in the opposite direction. They overlapped with sense genes or regulatory regions and could function both in cis or trans [9, 10]. Antisense lncRNAs could regulate protein-coding sense genes through the following mechanisms: transcriptional collision, gene recombination, promotor inactivation, alternative splicing, miRNA binding sites blocking, and endogenous siRNA formation [11–13]. For example, FOXP4-AS1 sponged miR-3184-5p to upregulate its host gene FOXP4 in prostate cancer [14]. SATB2-AS1 could cis-activate SATB2 transcription by decreasing the methylation level of SATB2 promoter through binding to WDR5 and GADD45A [15].

In ESCC, a few antisense lncRNAs that promoted or suppressed the tumorigenesis and progression of ESCC have been identified in previous studies. For example, silencing of ZEB1‐AS1 inhibited the expression of ZEB1 and suppressed ESCC progression [16]. EZR-AS1 could facilitate ESCC cell growth by positively regulating the expression of EZR via interacting with methyltransferase SMYD3 [17]. ZNF667-AS1 repressed ESCC cell proliferation and invasion by upregulating ZNF667 expression via interacting with TET1 and UTX, which could decrease histone H3K27 trimethylation to activate ZNF667 [18]. KLF3-AS1 functioned as a tumor suppressor in ESCC by sponging miR-185-5p and decreased its suppression on the expression of KLF3 [19]. All these studies suggested the vital roles of antisense lncRNAs and their sense genes in ESCC. However, no study has systematically evaluated the roles of antisense lncRNAs in ESCC progression.

Therefore, we performed a systematic evaluation on the roles and functions of antisense lncRNAs in the progression of ESCC. Firstly, differentially expressed antisense lncRNAs (Annotated using Gencode V29) were screened using transcriptome profiles from GSE53624 and were validated in 6 paired ESCC samples from our Jiangsu Province Hospital (JSPH, Jiangsu, China) database. Then, the log-rank test was adopted to assess the associations between promising antisense lncRNAs and ESCC prognosis. Finally, functional assays were carried out to reveal the functions of our identified antisense lncRNA.

## Results

### NNT-AS1 was aberrantly expressed in ESCC and was correlated with a poorer ESCC survival

**Fig. 1:**
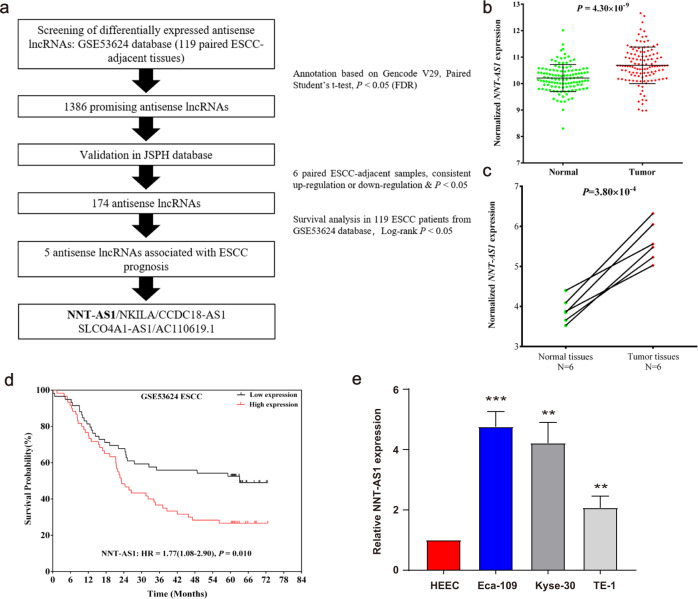
NNT-AS1 was aberrantly upregulated in ESCC and associated with poorer ESCC prognosis. **a** The screening process of NNT-AS; **b**, **c** NNT-AS1 expression was upregulated in ESCC tissues (**b** GSE53624, **c** JSPH, paired Student’s *t*-test); **d** high expression of NNT-AS1 was significantly associated with an inferior ESCC prognosis (Log-rank test); **e** the expression level of NNT-AS1 in ESCC cell lines (Eca-109, Kyse-30, and TE-1) was significantly higher than that in the human normal esophageal epithelial cell line (HEEC) (unpaired Student’s *t*-test). The results were from three independent experiments. ***P < 0.001, **P < 0.01. Variables were presented as the Mean ± SD (Standard Deviation).

Antisense lncRNAs annotation was performed based on Gencode V29. In total, 1386 promising antisense lncRNAs were initially screened in GSE53624. Among them, 174 lncRNAs were validated in our JSPH samples (Fig. S1). Five of them were significantly associated with ESCC prognosis: NNT-AS1, NKILA, CCDC18-AS1, SLCO4A1-AS1, AC11069.1 (Fig. 1a, S2, S3). Notably, NNT-AS1 showed the most robust correlation with the overall survival of ESCC patients. NNT-AS1 was abnormally expressed in ESCC tumor tissues in both GSE53624 (Fig. 1b) and JSPH databases (Fig. 1c). A higher expression of NNT-AS1 in ESCC patients indicated a worse prognosis (Fig. 1d). Likewise, NNT-AS1 expression was upregulated in ESCC cell lines compared with HEEC (Fig. 1e).

### Knockdown of NNT-AS1 suppressed ESCC progression

**Fig. 2:**
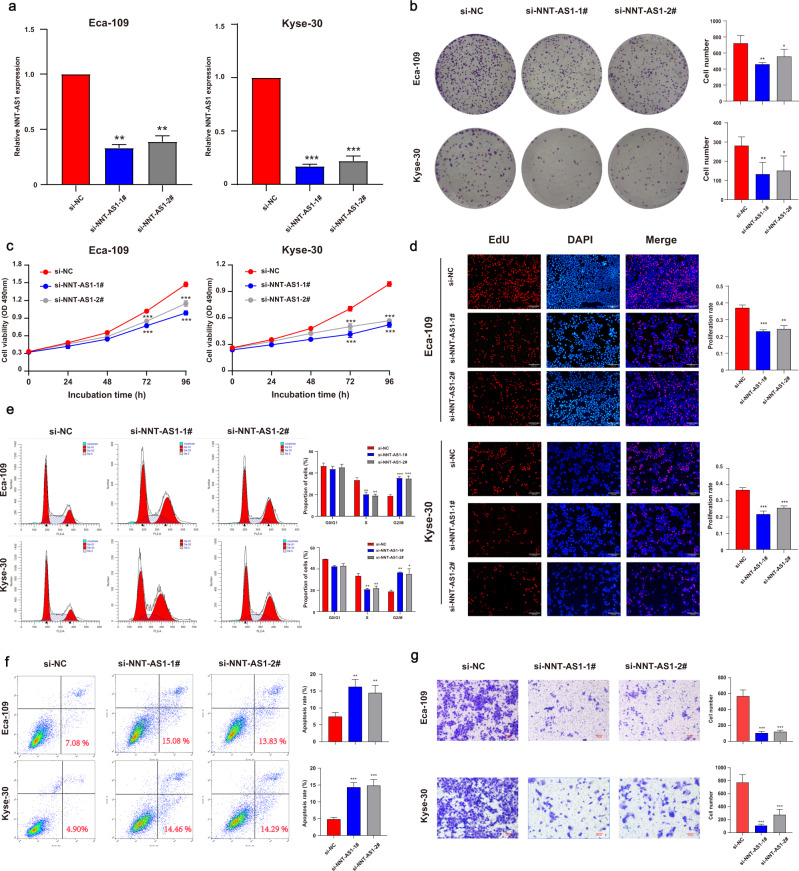
Knockdown of NNT-AS1 inhibited ESCC cell proliferation, migration, and induced cell cycle arrest, and promoted cell apoptosis. **a** The knockdown efficiency of short interfering RNAs for NNT-AS1; **b**–**d** knockdown of NNT-AS1 significantly inhibited the proliferation of Eca-109 and Kyse-30 cells using colony forming assays (**b**), MTT assays (**c**), and EdU assays (**d**); **e** cell cycle assays were employed to detect the distribution of cells in different phases; **f** knockdown of NNT-AS1 increased the apoptotic rate of both cell lines; **g** knockdown of NNT-AS1 inhibited the migration of Eca-109 and Kyse-30 cells. The results were from three independent experiments. Data were analyzed by unpaired Student’s *t*-test. ***P < 0.001, **P < 0.01, *P < 0.05. Variables were presented as the Mean ± SD.

To reveal the functions of NNT-AS1, two siRNAs were used to knock down NNT-AS1. The knockdown efficiency was shown in Fig. 2a. NNT-AS1 knockdown induced decreased colony formation (Fig. 2b) and suppressed ESCC cell viability (Fig. 2c). Likely, suppression of NNT-AS1 inhibited ESCC cell proliferation rate according to the EdU assays (Fig. 2d). Besides, NNT-AS1 inhibition induced the G2/M arrest in both Eca-109 and Kyse-30 cell lines (Fig. 2e). In addition, cell apoptosis assays demonstrated that the apoptosis rate of ESCC cells was increased after silencing NNT-AS1 (Fig. 2f). Furthermore, NNT-AS1 suppression inhibited the migration of ESCC cells (Fig. 2g). Taken together, NNT-AS1 knockdown restrained ESCC cell proliferation, migration, caused ESCC cell arrest, and facilitated cell apoptosis.

### NNT-AS1 positively regulated the expression of its sense gene NNT, which served as an oncogene in ESCC as well

**Fig. 3:**
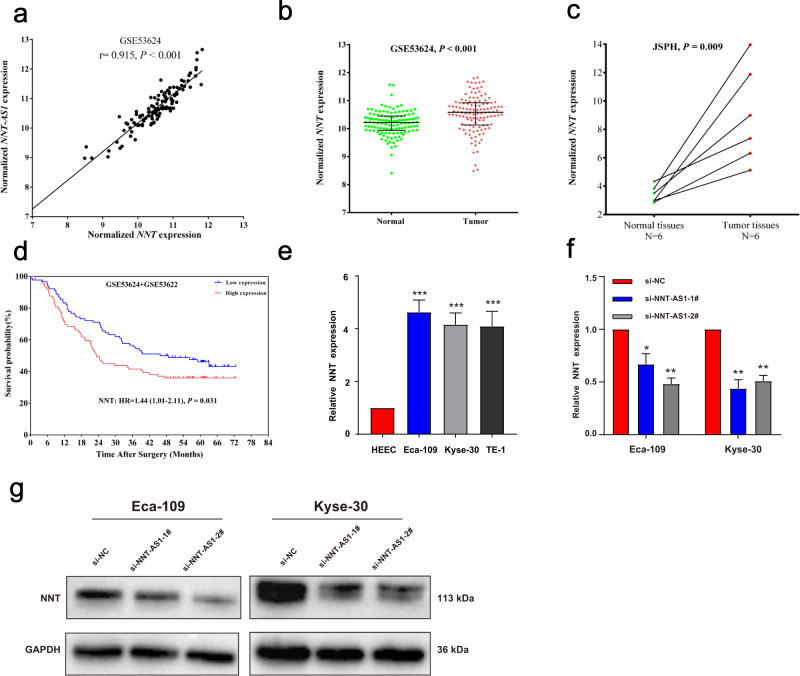
NNT was upregulated in ESCC and positively regulated by NNT-AS1. **a** NNT was positively correlated with NNT-AS1 in GSE53624 (*r* = 0.915, *P* < 0.001, Pearson Correlation); **b**, **c** NNT was upregulated in ESCC tissues (**b** GSE53624, *P* < 0.001; **c** JSPH, *P* = 0.009, paired Student’s *t*-test); **d** high expression of NNT was significantly associated with a poorer ESCC prognosis (Log-rank test); GSE53622 contained the mRNA profiles of 60 paired ESCC samples and was sequenced using the same platform with GSE53624; **e** the expression of NNT in three ESCC cells and HEEC cells (unpaired Student’s *t*-test); **f**, **g** knockdown of NNT-AS1 suppressed NNT in mRNA and protein levels (unpaired Student’s *t*-test). The results were from three independent experiments. ***P < 0.001, **P < 0.01, *P < 0.05. Variables were presented as the Mean ± SD.

Considering that NNT-AS1 was the antisense lncRNA of NNT, we wondered whether NNT-AS1 expression was correlated with that of NNT. As shown in Fig. 3a, NNT-AS1 expression significantly positively correlated with its sense gene NNT (*r* = 0.915, *P* < 0.001). NNT expression was also aberrantly upregulated in ESCC tumor tissues (Fig. 3b, c, GSE53624 and JSPH). Similarly, the high expression of NNT contributed to an inferior ESCC prognosis in combined datasets of GSE53624 and GSE53622 (Fig. 3d, HR = 1.44 (1.01-2.11), *P* = 0.031). Consistent with tissue samples, NNT was notably upregulated in ESCC cells (Fig. 3e). To demonstrate the influence of NNT-AS1 on NNT expression, we knocked down NNT-AS1 and detected the NNT expression. As expected, NNT-AS1 silencing significantly suppressed NNT expression in mRNA level (Fig. 3f), as well as in the protein level **(**Fig. 3g), suggesting that NNT-AS1 could regulate its sense gene NNT expression.

**Fig. 4:**
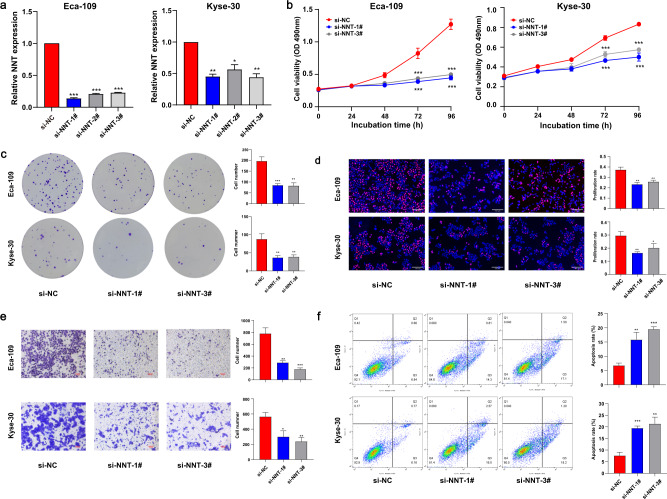
Knockdown of NNT suppressed ESCC cell proliferation and migration. **a** The knockdown efficiency of short interfering RNAs for NNT was measured in Eca-109 and Kyse-30; **b**–**d** Knockdown of NNT inhibited proliferation of Eca-109 and Kyse-30 cells using MTT assays, colony forming assays, and EdU assays; **e** knockdown of NNT inhibited the migration of Eca-109 and Kyse-30 cells; **f** Knockdown of NNT increased the apoptotic rate of both ESCC cell lines. The results were from three independent experiments. Data were analyzed by unpaired Student’s *t*-test. ***P < 0.001, **P < 0.01, *P < 0.05. Variables were presented as the Mean ± SD.

To reveal the functions of NNT in ESCC, three siRNAs were synthesized to knock down the expression of NNT (Fig. 4a). MTT, colony formation, and EdU assays indicated that NNT knockdown significantly inhibited the proliferation of ESCC cells (Fig. 4b–d). Transwell assay showed that inhibition of NNT suppressed ESCC cell migration (Fig. 4e). Furthermore, inhibition of NNT increased the apoptosis rate of ESCC cells (Fig. 4f). All the findings revealed that NNT functioned as a carcinogene in the tumorigenesis and development of ESCC.

### NNT-AS1 could sponge the miR-382-5p in ESCC

**Fig. 5:**
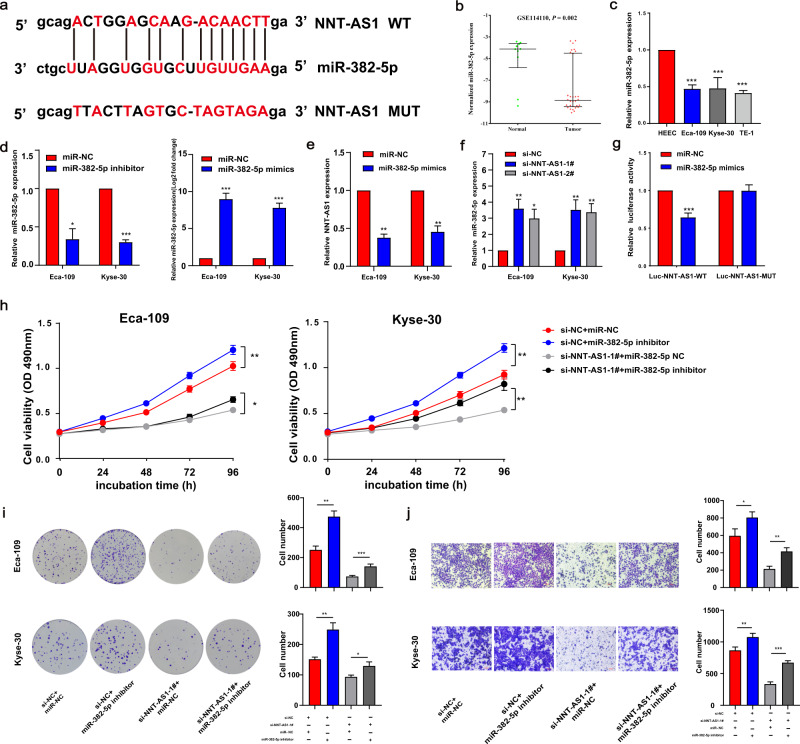
NNT-AS1 could sponge miR-382-5p and knockdown of miR-382-5p could rescue the decreased ESCC proliferation and migration mediated by NNT-AS1 inhibition. **a** MiR-382-5p had a binding site for NNT-AS1; **b** MiR-382-5p was downregulated in ESCC tissues based on GSE114110 (*P* = 0.002); **c** the expression of miR-382-5p in three ESCC cells and HEEC; **d** the transfection efficiency of miR-382-5p mimics and inhibitor were verified; **e** overexpression of miR-382-5p inhibited the NNT-AS1 expression; **f** knockdown of NNT-AS1 increased miR-382-5p expression; **g** Dual-Luciferase assays detected the luciferase activities in Eca-109 cells after being co-transfected with miRNA (miR-NC or miR-mimics) and luciferase reporter vectors (NNT-AS1-WT or NNT-AS1-MUT); **h**–**j** MiR-382-5p partly retrieved the suppressed abilities of proliferation (**h** MTT; **i** colony formation) and migration (**j**) that NNT-AS1 knockdown mediated in ESCC cell lines. The results were from three independent experiments. Data were analyzed by unpaired Student’s *t*-test. ***P < 0.001, **P < 0.01, *P < 0.05. Variables were presented as the Mean ± SD.

LncRNA-miRNA-mRNA axis was a crucial mechanism through which lncRNAs influenced the tumorigenesis and progression of malignant tumors [20, 21]. We predicted the NNT-mediated miRNA using the ENCORI database, and 49 miRNAs were screened. Subsequently, we analyzed the differentially expressed miRNAs based on the GSE114110 database. As a result, six of them were markedly downregulated, including miR-382-5p, miR-26a-5, miR-26b-5p, miR-186-5p, miR-130a-3p, and miR-582-5p. Among them, the foldchange of miR-382-5p ranks first. Additionally, a previous investigation manifested that miR-382 could suppress tumor progression against ESCC [22]. Thus, we hypothesized that NNT-AS1 might regulate NNT expression through sponging miR-382-5p. Figure 5a illustrated the predicted binding site of NNT-AS1 and miR-382-5p by Starbase. As anticipated, miR-382-5p expression was apparently decreased in ESCC (Fig. 5b, c).

Figure 5d showed the transfection efficacies of miR-382-5p mimics and inhibitors. The miR-382-5p mimics significantly inhibited NNT-AS1 expression (Fig. 5e). Knockdown of NNT-AS1 led to increased miR-382-5p expression (Fig. 5f). Further, dual-luciferase reporter assays indicated that miR-382-5p overexpression weakened the luciferase activity in the NNT-AS1-WT group (Fig. 5g). Furthermore, the rescue assays demonstrated that miR-382-5p inhibition partially rescued the declined proliferation and migration abilities of ESCC cells mediated by NNT-AS1 knockdown (Fig. 5h–j).

### NNT was regulated by miR-382-5p and retrieved the decreased cell functions mediated by NNT-AS1 silence

**Fig. 6:**
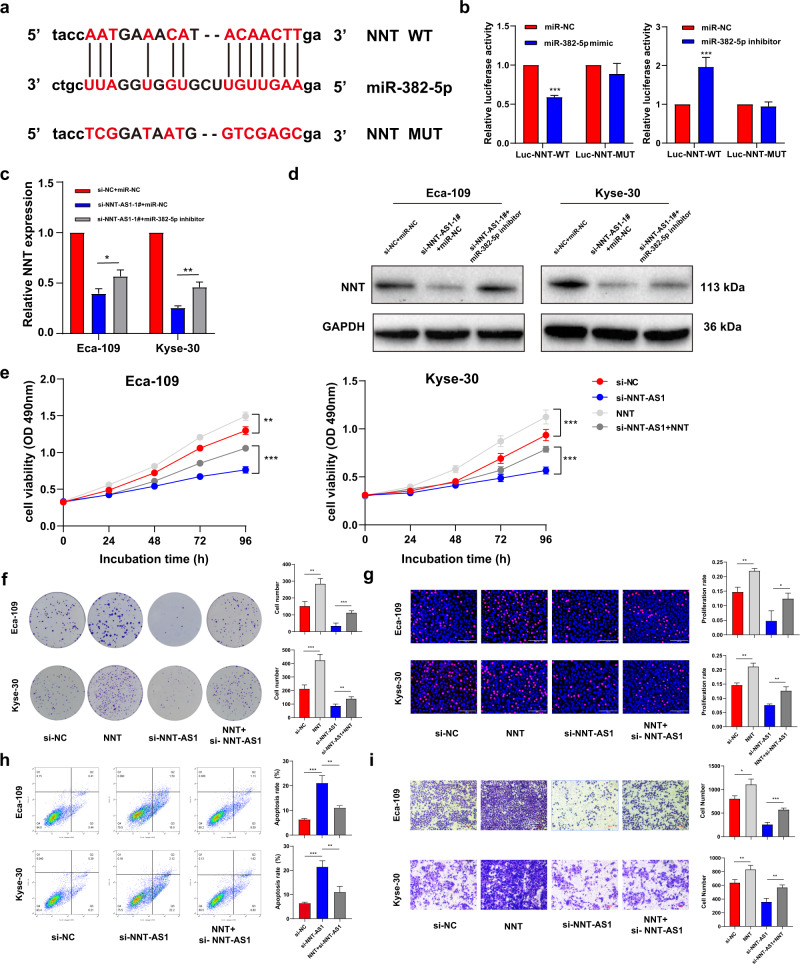
MiR-382-5p negatively regulated NNT and overexpression of NNT rescued the decline of cellular function that NNT-AS1 knockdown mediated in ESCC. **a** MiR-382-5p had a binding site for NNT; **b** Dual-Luciferase assays were utilized to detect the luciferase activities in Eca-109 cells after being co-transfected with miRNAs (miR-NC, miR-382-5p mimics, miR-382-5p inhibitor) and luciferase reporter vectors (NNT- WT and NNT- MUT) respectively; **c**, **d** downregulation of miR-382-5p rescued the decreased NNT expression mediated by NNT-AS1 inhibition in Eca-109 and Kyse-30 cells; **e**–**i** Rescue assays were conducted to measure the cellular functions of NNT-AS1 knockdown cells after overexpressing NNT in Eca-109 and Kyse-30 cells. The results were from three independent experiments. Data were analyzed by unpaired Student’s *t*-test. ***P < 0.001, **P < 0.01, *P < 0.05. Variables were presented as the Mean ± SD.

The predicted binding locus of miR-382-5p and NNT was shown in Fig. 6a. Co-transfection with NNT-WT and miR-382-5p mimics in Eca-109 weakened the luciferase activity compared to NNT-WT and miR-NC, while NNT-Mut failed to induce such a reduction. In contrast, NNT-WT and miR-382-5p inhibitor co-transfection led to increased luciferase activity in comparison with the miR-NC (Fig. 6b). Further, inhibition of miR-382-5p partly retrieved the decreased NNT expression in both mRNA and protein levels induced by NNT-AS1 knockdown (Fig. 6c, d). NNT overexpression facilitated ESCC cell proliferation and migration. As expected, overexpression of NNT could countervail the suppressed ESCC cell proliferation induced by NNT-AS1 inhibition (Fig. 6e–g). Furthermore, NNT rescued the increased apoptosis rate generated by NNT-AS1 suppression (Fig. 6h). NNT upregulation also recovered cell migration that declined by NNT-AS1 knockdown (Fig. 6i). Taken together, NNT-AS1 facilitated ESCC tumorigenesis and process by modulating its sense gene NNT expression.

### NNT-AS1 promoted ESCC cell growth in vivo by regulating the cell cycle signaling pathway

**Fig. 7:**
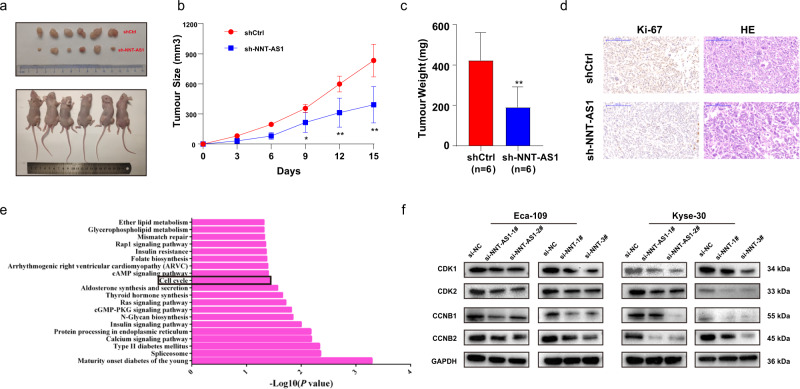
NNT-AS1 promoted cell growth in vivo and could regulate the cell cycle signaling pathway. **a** ShCtrl or shNNT-AS1 was stably transfected into Eca-109 cells, which were respectively injected into the back of nude mice; **b** tumor volumes were measured every 3 days and the growth curve of tumors was plotted; **c** the tumor weight in the shCtrl group was higher than that in the shNNT-AS1 group; **d** the tumor sections were undergone ki-67 staining and HE staining; **e** pathway enrichment analysis was conducted to predict the potential pathways that NNT participated in ESCC; **f** several checkpoints of cell cycle pathway were measured using western blot assays after silencing NNT and NNT-AS1. The results were from three independent experiments. Data were analyzed by unpaired Student’s *t*-test. ***P < 0.001, **P < 0.01, *P < 0.05. Variables were presented as the Mean ± SD.

The role that NNT-AS1 played in the tumor growth of ESCC was further evaluated by the xenograft model. As shown in Fig. 7a, b, mice with Eca-109 cells stably transfected with shNNT-AS1 had a smaller tumor volume than the shCtrl group. In line, compared to mice in the shCtrl arm, those in the shNNT-AS1 group exhibited a lower tumor weight (Fig. 7c). Moreover, the Ki-67 positivity was markedly decreased in tumors formed from Eca-109 cells transfected with shNNT-AS1 (Fig. 7d). All the findings suggested that the silence of NNT-AS1 could suppress ESCC tumor growth in vivo.

To suggest the potential down-stream pathway through which NNT contributed to ESCC tumorigenesis and progression, pathway enrichment analysis was performed. As shown in Fig. 7e, NNT might participate in multiple signaling pathways, including the cell cycle pathway, one of the central pathways in cancers. This result was consistent with the above flow cytometry findings. Western blot assays indicated that NNT-AS1 and NNT silencing could suppress the levels of CCNB1, CCNB2, CDK1, and CDK2 (Fig. 7f). Taken together, NNT-AS1 contributed to ESCC initiation and development through regulating the cell cycle pathway.

## Discussion

In this study, we evaluated the roles of antisense lncRNAs in ESCC through a systematic analysis. As a result, NNT-AS1 was found aberrantly upregulated in ESCC and was significantly associated with a poorer prognosis. Functionally, we found that NNT-AS1 might contribute to ESCC progression through positively regulating its sense gene NNT expression by sponging miR-382-5p.

NNT-AS1, located in 5p12, has been verified to play a carcinogenic role in multiple malignant tumors, including lung squamous cell carcinoma, cervical cancer, gastric cancer, and so on. Ma *et al* discovered that NNT-AS1 accelerated the progression of lung squamous cell carcinoma by positively modulating FOXM1, a member of the FOX transcription factor family [23]. Li *et al* revealed that NNT-AS1 facilitated breast cancer progression by regulating ZEB1 expression through sponging miR-142-3p [24]. Chen and colleagues demonstrated that NNT-AS1 could contribute to the tumorigenesis of gastric cancer by targeting E2F1 which served as a transcription factor and was an important regulator in cell cycle [25]. Nevertheless, the functions and roles of NNT-AS1 in ESCC remained unclear. NNT-AS1 was found aberrantly expressed in ESCC and showed a significant association with a poorer survival of ESCC for the first time. Functionally, silencing of NNT-AS1 restrained ESCC cell proliferation and migration. Besides, NNT-AS1 inhibition could induce the arrest of G2/M phase in cell cycle progression and promot ESCC cell apoptosis. Tumor xenograft experiment in nude mice indicated that silencing of NNT-AS1 slackened the tumor growth in *vivo*. All these results manifested that NNT-AS1 could promote the tumorigenesis and progression of ESCC. However, which was the target gene of NNT-AS1, and what was the molecular mechanism underlying NNT-AS1’s functions in ESCC?

Considering that NNT was the sense gene of NNT-AS1 and regulating the sense gene expression was a vital mechanism through which antisense lncRNAs modulated tumorigenesis and progression, we analyzed the correlation ship between NNT-AS1 and NNT. Strikingly, the expression of NNT was significantly correlated with NNT-AS1 expression, suggesting that NNT-AS1 might regulate NNT expression. As expected, knockdown of NNT-AS1 significantly suppressed NNT expression. Functionally, NNT encoded an integral protein of the inner mitochondrial membrane. Previous studies showed that NNT served as an oncogene in multiple cancers. For example, knockdown of NNT significantly suppressed gastric cancer growth and metastasis through the oxidative stress pathway [26]. NNT could regulate mitochondrial metabolism in lung cancer by maintaining the function of the Fe-S protein [27]. Nevertheless, the roles of NNT in ESCC progression have not been revealed. In this study, we observed that the silence of NNT exerted inhibitory effects on the growth and migration of ESCC cells, while NNT overexpression partially retrieved the decreased proliferation and migration abilities induced by NNT-AS1 inhibition. Highlighting these findings, we speculated that NNT-AS1 could modulate ESCC tumorigenesis and progression by regulating its sense gene NNT.

Given that ceRNA was a common mechanism through which lncRNAs regulated the target genes [28, 29], we predicted the potential miRNAs that could interact with NNT-AS1. Consequently, miR-382-5p was determined as the candidate miRNA due to its potential of binding to NNT-AS1 and aberrant expression in ESCC. Through dual-luciferase assays, we verified that miR-382-5p could combine with NNT-AS1 and NNT. MiR-382-5p inhibition retrieved the decreased NNT expression that NNT-AS1 knockdown mediated in ESCC cell lines. Functionally, rescue experiments further validated that miR-382-5p suppression reversed the inhibitory impacts of NNT-AS1 knockdown on ESCC progression. Additionally, miR-382-5p was reported to suppress ESCC progression and was associated with a favorable prognosis in previous studies [22, 30]. All the findings above suggested that NNT-AS1 acted as a ceRNA by sponging miR-382-5p to regulate its sense gene NNT.

The cell cycle signaling pathway was crucial in the development of malignant tumors [31, 32]. This study found that NNT-AS1 knockdown induced the G2/M arrest in ESCC cell lines. Besides, pathway enrichment analysis indicated that NNT might participate in the modulation of the cell cycle signaling pathway in ESCC. Western blot assays further confirmed that inhibition of NNT and NNT-AS1 suppressed the expression levels of CDK1, CDK2, CCNB1, and CCNB2, all of which were vital proteins in the cell cycle signaling pathway. Taken together, these findings suggested that NNT-AS1 and NNT could promote ESCC progression by regulating the cell cycle signaling pathway.

## Conclusions

Antisense lncRNA NNT-AS1 promoted ESCC progression by targeting its sense gene NNT through competitively sponging miR-382-5p. This study provided us with a deeper insight into the roles of antisense lncRNAs in ESCC and identified novel potential therapeutic targets.

## Materials and methods

### Tissue samples and transcriptome sequencing

Six paired ESCC tumor-normal tissues were obtained from JSPH. Total RNA was extracted from the samples. Then ribosomal RNAs were removed by TruSeq StrandedTotal RNA with Ribo-Zero Gold Kit (Illumina, San Diego, California, USA), and the residual RNAs were broken into short fragments. After purification, the end of cDNA was repaired and poly-A tail was added as well as the sequencing connector was connected to the cDNA. Finally, the RNA library was established by PCR amplification and assessed by Agilent2100Bioanalyzer. The sequencing was performed using the Illumina sequenator (Illumina, San Diego, California, USA). The DESeq2 software was used for the normalization and differential expression analysis. The ethics committee of the First Affiliated Hospital of Nanjing Medical University approved this study. All the patients signed the informed consent.

### Annotation and screening of antisense lncRNAs

The GENCODE project provided comprehensive and detailed annotations for the human and mouse genomes [33]. In this study, we obtained the lncRNA annotation from GENCODE (Gencode V29, https://www.gencodegenes.org/). There were 5587 lncRNAs annotated as antisense lncRNAs according to the Gencode V29. The GSE53624 dataset that contained lncRNA and mRNA expression profiles of 119 paired ESCC tumor-normal samples was used for differential antisense lncRNAs screening. After annotation, 2003 antisense lncRNAs were identified in GSE53624. Among them, 1386 lncRNAs were significantly differentially expressed in ESCC (Paired Student’s *t*-test, *P* < 0.05, false discovery rate (FDR) correction).

### Cell culture and transfection

Human ESCC cell lines (Kyse-30, Eca-109, and TE-1) and human normal esophageal epithelial cell line (HEEC) were purchased from iCell Bioscience, Shanghai, China. RPMI-1640 medium (Gibco, Rockville, Maryland, USA) with 10% fetal bovine serum (FBS; Biological Industries, Israel), penicillin-G (100 U/ml), and streptomycin (100 g/ml) (Gibco, Rockville, Maryland, USA) was used for Kyse-30 and TE-1 incubation. DMEN (Gibco, Rockville, Maryland USA) contains the same ingredients were used for Eca-109 and HEEC. The cultivation environment was man-made at 37 °C with 5% CO_2_. The sequence of transfected RNAs together with the name of suppliers were listed in Table S1. For overexpression of NNT, the cDNA encoding NNT was amplified and cloned into the pcDNA3.1 vector (Invitrogen, Carlsbad, New Mexico, USA) to form NNT overexpression plasmid. Transfections were carried out using Lipofectamine 3000 (Invitrogen, Carlsbad, CA, USA) following instructions.

### Quantitative Real‐time PCR (qRT-PCR)

The cell lines at the period of logarithmic growth were transfected. Then the total RNA was extracted using TRIzol reagent (Invitrogen, Carlsbad, CA, USA) 48 h later. The extracted RNAs were reversely transcribed to cDNAs using PrimeScriptTM II Reverse Transcriptase (Takara, Tokyo, Japan). Gene expression was calculated as previously reported [34]. Primer sequences were illustrated in Table S1.

### MTT assay

2 × 10^3^ transfected cells were planted in a 96-well plate per well. At different checking points (0, 24, 48, 72, and 96 h), the cells were incubated in a complete medium with 20 μL MTT (concentration: 0.5 mg/mL) for 4 h. Then DMSO (150 μL per well) was used to dilute the formazan that formed from MTT. After 10 min concussion, the cell viability was measured at the absorbance of 490 nm.

### Colony forming assay

2 × 10^3^ transfected cells were planted in a 6-well plate per well and incubated in a complete medium until the colonies could be detected by naked eyes. Then the colonies were cleaned with normal saline and fixed with 4% paraformaldehyde (room temperature, 15 min) followed by staining using 0.5% crystal violet staining (Beyotime, Shanghai, China). ImageJ software was used to detect the number of colonies.

### 5‑Ethynyl‑2′‑deoxyuridine (EdU) assay

A total of 1 × 10^5^ transfected cells were added evenly in 96-well plate per well and incubated for two days. EdU assays were performed using YF®555 Click-iT EdU Imaging Kits (US EVERBRIGHT, China) under the instruction of the manufacturer. The treated cells were detected under an inverted fluorescence Microscope and the graphs were captured simultaneously from three random fields. The proliferation rate in EdU staining was defined as the percentage of EdU staining cells relative to cells stained by DAPI.

### Transwell assay

Transwell assays were carried out using Transwell apical chamber (Corning Life Sciences, Corning, NY, USA). In total, 5 × 10^4^ transfected cells were added into the upper compartment, while 700 µl complete medium was injected into the lower part. After 24 h, cells were fixed using 4% paraformaldehyde followed by staining with 0.5% crystal violet. Then, cells that failed to migrate to the outer surface of the upper compartment were scrubbed by cotton swabs. Five microscopic fields per chamber were observed for counting the migratory cells (at ×100 magnification). The count of migratory cells was performed using ImageJ software.

### Flow cytometry assays

A total of 5 × 10^5^ cells were cultured and transfected. 48 h after transfection, the collected adherent cells were fixed in 75% ethanol followed by staining with PI. Cell apoptosis assay was carried out according to the protocol of YF®488-Annexin V and PI Apoptosis Kit (US EVERBRIGHT, China). The results were assessed by FACSCalibur (BD, Franklin Lakes, NJ, USA). FlowJo software 10.8.0 (BD, Franklin Lakes, NJ, USA) was used to perform the analysis.

### Dual-luciferase assay

The potential binding sites of miR-382-5p in NNT-AS1 and NNT were predicted by Starbase. The binding sites were cloned in the pmirGLO vector (Promega, Madison, WI, USA) to generate luciferase reporter vectors of wild type (NNT-AS1 WT, NNT WT) and corresponding mutant type vectors (NNT-AS1 MUT, NNT MUT). Lipofectamine 3000 (Invitrogen, Carlsbad, CA, USA) was used for co-transfection. The Dual-Luciferase Reporter Assay Kit (Vazyme, Nanjing, Jiangsu, China) was used for luciferase assays according to the protocols.

### Western blot assay

Proteins were extracted and quantified using RIPA lysis buffer (Beyotime, Shanghai, China) and BCA Protein Assay kit (Thermo Fisher Scientific, Waltham, MA, USA), respectively. The proteins with loading buffer (volume ratio=1:4) were added in lanes of 4–20% SurePAGE (GeneSript, Nanjing, Jiangsu, China). After electrophoresis, the protein gel was transferred to the PVDF membrane (Millipore, Massachusetts, USA) and blocked using 5% skimmed milk in TBST. Then, the membranes were incubated in specific antibodies as follows: GAPDH (AC001, 1:2000) NNT (A4561, 1:500), CDK1 (A0220, 1:1000), CDK2 (A18000, 1:1000), CCNB1 (A19037, 1:1000), and CCNB2 (A3351, 1:1000). All the antibodies mentioned above were purchased from Abcolonal, Wuhan, China. GelDoc XR + (Bio-Rad, Hercules, CA, USA) was adopted to develop the protein bands.

### Animal experiment

Stably transfected Eca-109 cells with NNT-AS1 silence (shNNT-AS1) and negative control (shCtrl) were constructed for animal experiments. The sequence of shNNT-AS1 was illustrated in Table S2. In brief, Eca-109 cells were infected with lentivirus-coated shNNT-AS1 and shCtrl, which contained the green fluorescent protein. The transfection efficiency was assessed through observing the fluorescence intensity under a fluorescence microscope and qRT-PCR was utilized for accurate evaluation.

Six 6 weeks old nude male mice were purchased from Weitonglihua, Beijing, China. The stably transfected Eca-109 cells were inoculated into the back of the mice, right for shNNT-AS1 and left for shCtrl respectively. The diameter of the tumor was measured and recorded every 3 days. The volume of the tumor was determined as: V = LD × (SD)^2^/2 (LD: the largest diameter; SD: the shortest diameter). The nude mice were euthanized on the 15^th^ day through cervical dislocation and the tumors were dissected and weighed. All animal experiments were performed under the protocols approved by the Experimental Animals Ethics Committee of Nanjing Medical University.

### Statistical analysis

In this study, paired student’s *t*-test was adopted for the comparison of lncRNA expression in ESCC tumor and adjacent normal tissues. The false discovery rate (FDR) was used to correct the *P* values. Associations between lncRNA expression and ESCC prognosis were assessed using the Log-rank test. The unpaired student’s *t*-test was applied to test the differences between experimental groups. The expression relationship between NNT-AS1 and NNT was assessed based on Pearson correlation. Statistical tests were conducted based on R 3.6.0 and Graphpad Prism 8.0. The statistical significance level was set at *P* < 0.05.

## Supplementary information

Supplementary Tables

Supplementary Figure

Original Data File

## Data Availability

The datasets used and analyzed in this study are available from the corresponding author on reasonable request.
