# Supplementary material for: Antisense lncRNA NNT-AS1 promoted esophageal squamous cell carcinoma progression by regulating its sense gene NNT expression
Source: Cell Death Discov. 2022 Oct 21;8:424. doi: 10.1038/s41420-022-01216-w (PMC9586939; doi:10.1038/s41420-022-01216-w)
Supplement: Supplementary file 1 — Supplementary Tables [file 41420_2022_1216_MOESM1_ESM.docx]

**Table S1. The sequences of siRNAs, miRNAs, and primers used in this study.**

|  | **Sequences** | **Supplier** |
| --- | --- | --- |
| si-NNT-AS1-1# | sense: CAUCUGCUUCUGGUGACAACCUCAATT | PROTEINBIO, Nanjing, China |
|  | antisense: UUGAGGUUGUCACCAGAAGCAGAUG |  |
| si-NNT-AS1-2# | sense: GAAGGACAGACAAACUGUAACCUUUTT |  |
|  | antisense: AAAGGUUACAGUUUGUCUGUCCUUCTT |  |
| si-NNT -1# | sense: GCACCUUUGUUGGUGGAUAUUT |  |
|  | antisense: AAUAUCCACCAACAAAGGUGCTT |  |
| si-NNT -2# | sense: CCCUAUGGUUAAUCCAACAUUTT |  |
|  | antisense: AAUGUUGGAUUAACCAUAGGGTT |  |
| si-NNT -3# | sense: CGAGAAGCUAAUAGCAUUAUUTT |  |
|  | antisense: AAUAAUGCUAUUAGCUUCUCGT |  |
| miR-382-5p mimics | forward: GAAGUUGUUCGUGGUGGAUUGG | TSINGKE, Nanjing, China |
|  | reverse: AAUCCACCACGAACAACUUCUU |  |
| mimics NC | forward: CTCGCTTCGGCAGCACA |  |
|  | reverse: AACGCTTCACGAATTTGCGT |  |
| miR-382-5p inhibitor | CGAAUCCACCACGAACAACUUC |  |
| inhibitor NC | CAGUACUUUUGUGUAGUACAA |  |
| NNT-AS1 forward | ACGTGCAGACAACATCTACCT | GENERAY, Shanghai, China |
| NNT-AS1 reverse | TACAACACCTTCCCGCAT |  |
| NNT forward | TGGTCAAGCAGGGTTTTAATGT |  |
| NNT reverse | TCCTTTGCCCCTTGGATTTGG- |  |
| miR-382-5p forward | ATCCGTGAAGTTGTTCGTGG |  |
| miR-382-5p reverse | TATGGTTGTAGAGGACTCCTTGAC |  |
| GAPDH forward: | GAAGTGTGAAGGTCGGAGTC |  |
| GAPDH reverse | GAAGATGGTGATGGGATTTC |  |

**Table S2**. **The sequence of shNNT-AS1.**

|  | **5’** | **stem** | **loop** | **stem** | **3’** |
| --- | --- | --- | --- | --- | --- |
| shNNT-AS1-a | Ccgg | CATCTGCTTCTGGTGACAACCTCAA | CTCGAG | TTGAGGTTGTCACCAGAAGCAGATG | TTTTTg |
| shNNT-AS1-b | aattcaaaaa | CATCTGCTTCTGGTGACAACCTCAA | CTCGAG | TTGAGGTTGTCACCAGAAGCAGATG |  |
