# Supplementary material for: Antisense lncRNA NNT-AS1 promoted esophageal squamous cell carcinoma progression by regulating its sense gene NNT expression
Source: Cell Death Discov. 2022 Oct 21;8:424. doi: 10.1038/s41420-022-01216-w (PMC9586939; doi:10.1038/s41420-022-01216-w)
Supplement: Supplementary file 2 — Supplementary Figure [file 41420_2022_1216_MOESM2_ESM.docx]

**Supplementary Information**

**Figure S1**: Differentially expressed anti-sense lncRNAs in GSE53624 and JSPH.

**Figure S2**: Expression of AC11069.1, CCDC18-AS1, NKILA, and SLCO4A1-AS1 in ESCC tumor and adjacent normal tissues in GSE53624 and JSPH databases.

**Figure S3**: Associations between four anti-sense lncRNAs and ESCC prognosis.


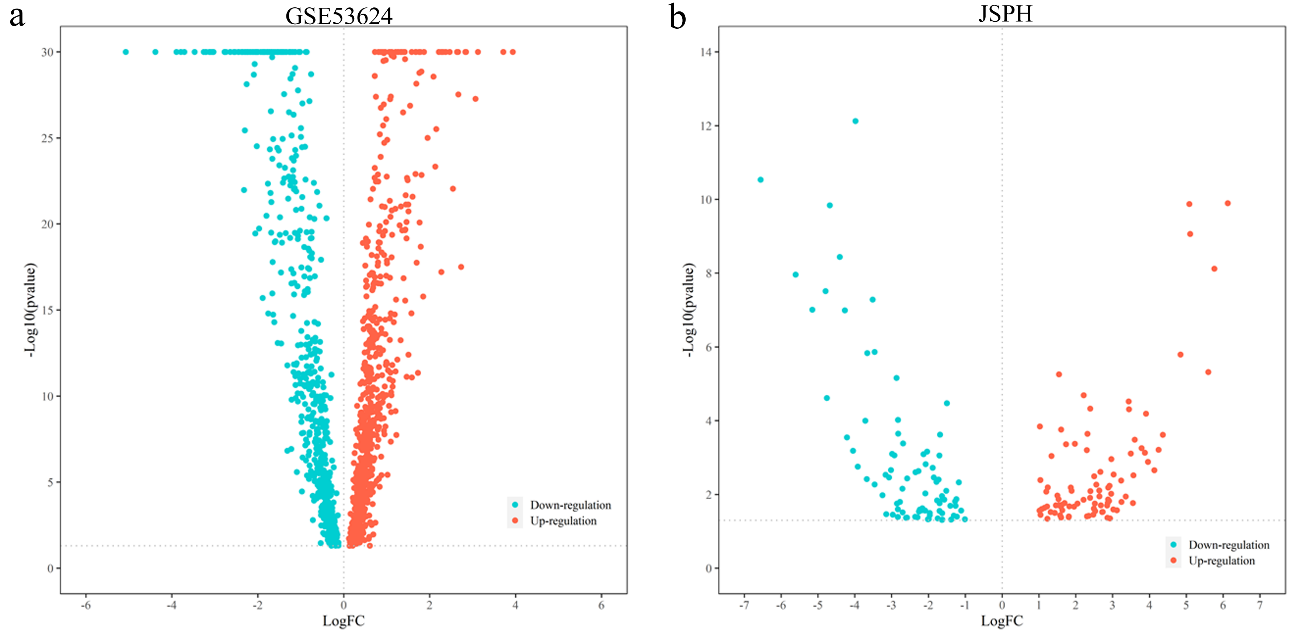
 **Figure S1.** **Differentially expressed anti-sense lncRNAs in GSE53624 (a) and JSPH (b).**


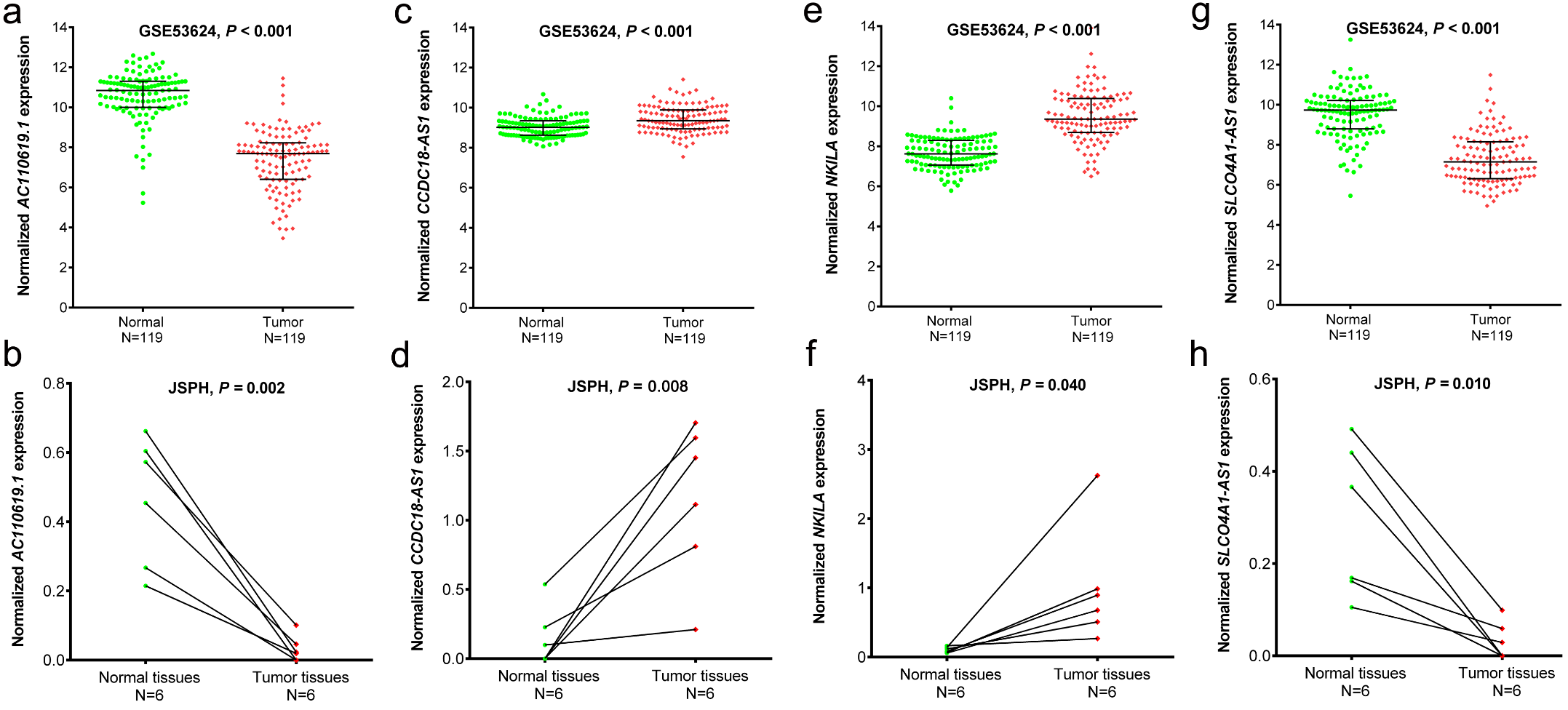


**Figure S2. Expression of AC11069.1, CCDC18-AS1,** **NKILA and SLCO4A1-AS1 in ESCC tumor and adjacent normal tissues in GSE53624 and JSPH databases.**

a-b: AC11069.1 was aberrantly down-regulated in ESCC tumor tissues (a: GSE53624; b: JSPH); c-d: The expression of CCDC18-AS1 in ESCC tumors was significantly increased compared to that in paired normal tissues (c: GSE53624; d: JSPH); e-f: The expression of NKILA in ESCC tumors was significantly higher than that in paired normal tissues (e: GSE53624; f: JSPH); g-h: SLCO4A1-AS1 was aberrantly down-regulated in ESCC tumor tissues (g: GSE53624; h: JSPH); Paired Student’s t-test was used for differential expression analysis.


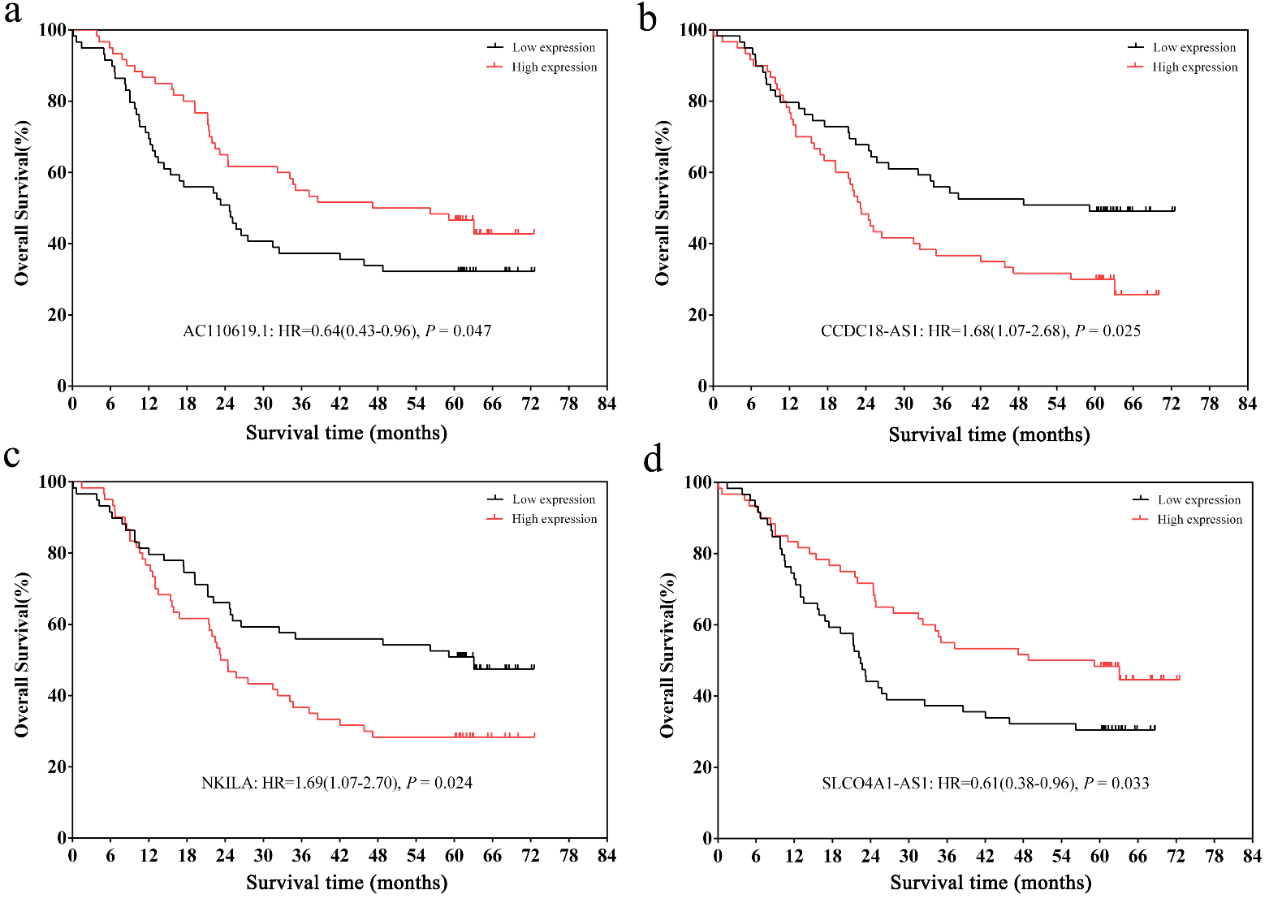


**Figure S3. Associations between four anti-sense lncRNAs and ESCC prognosis.**

a: ESCC patients with a higher AC11069.1 expression had a better prognosis than those with a lower AC11069.1; b: High expression of CCDC18-AS1 was significantly associated with a worse ESCC prognosis; c: ESCC patients with a higher NKILA expression had a poorer prognosis than those with a lower NKILA expression; d: High expression of SLCO4A1-AS1 showed a significant association with a more favorable ESCC prognosis; Survival analysis was performed based on GSE53624 database using the Log-rank test.
