# Supplementary figures and images for: Antisense lncRNA NNT-AS1 promoted esophageal squamous cell carcinoma progression by regulating its sense gene NNT expression
Source: Cell Death Discov. 2022 Oct 21;8:424. doi: 10.1038/s41420-022-01216-w (PMC9586939; doi:10.1038/s41420-022-01216-w)

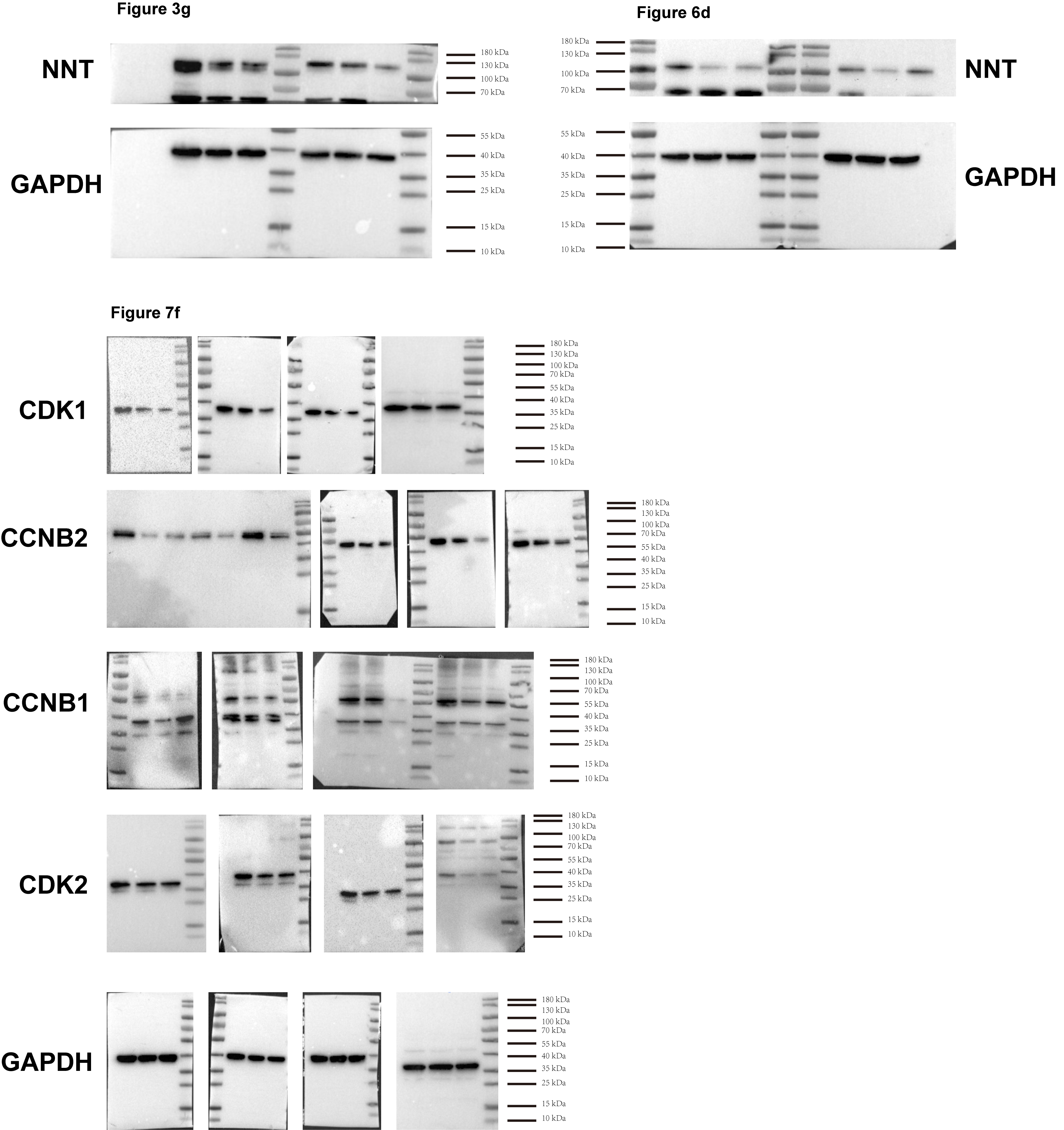

Supplement: Supplementary file 3 — Original Data File [file 41420_2022_1216_MOESM3_ESM.tif]
